# IP-10, MCP-1, MCP-2, MCP-3, and IL-1RA hold promise as biomarkers for infection with *M. tuberculosis *in a whole blood based T-cell assay

**DOI:** 10.1186/1756-0500-2-19

**Published:** 2009-02-04

**Authors:** Morten Ruhwald, Morten Bjerregaard-Andersen, Paulo Rabna, Jesper Eugen-Olsen, Pernille Ravn

**Affiliations:** 1 Dep. of Infectious Diseases 144, Copenhagen University, Hvidovre Hospital, DK-2650 Hvidovre, Denmark; 2 Clinical Research Centre 136, Copenhagen University, Hvidovre Hospital, DK-2650 Hvidovre, Denmark; 3 Dep. of Infectious Diseases Q, Skejby University Hospital, 8200 Aarhus N, Denmark; 4 Bandim Health Project, Apartado 861, 1004 Bissau Codex, Guinea Bissau; 5 Department of Medicine, Unit for Infectious Diseases, Copenhagen University, Herlev Hospital, 2730 Herlev, Denmark

## Abstract

**Background:**

IFN-γ responses to *M. tuberculosis *antigens are used as *in-vitro *diagnostic tests for tuberculosis infection. The tests are highly specific but sensitivity may be impaired due to immuno-suppression. The objective of this small exploratory study was to compare three novel biomarkers for *in-vitro *diagnosis of tuberculosis – MCP-1, MCP-3 and IL-1RA – with the current established biomarker IFN-γ and the newly described IP-10 and MCP-2.

**Methods:**

Whole blood from 8 patents with active tuberculosis and from 7 healthy controls was stimulated with *M. tuberculosis *specific antigens and mitogen in the Quantiferon In Tube test tubes. Levels of biomarkers were measured using Luminex and ELISA (IFN-γ).

**Results:**

We found all five new biomarkers were expressed in significantly higher concentrations compared to IFN-γ. IP-10 and MCP-3 levels in the un-stimulated samples were higher in patients compared with controls.

**Conclusion:**

All biomarkers had diagnostic potential as they could differentiate between the patients and the controls. IP-10 and MCP-2 seemed most promising as they were expressed in high levels with antigen stimulation and were low in the un-stimulated samples. Further studies are needed to explore the potential of these highly expressed novel biomarkers individually and in combination.

## Background

Until recently the tuberculin skin test has been the key tool in the diagnosis of latent tuberculosis infection, but with the identification of three *Mycobaterium tuberculosis *(TB) specific protein antigens (ESAT-6, CFP10, and TB 7.7) a new generation of diagnostic tests has emerged. These tests measure interferon gamma (IFN-γ) release after the stimulation of whole blood or blood components with the antigens and are known as Interferon Gamma Release Assays (IGRA). The tests have proved to be both sensitive and specific for the detection of TB infection[1]. However, new reports suggest that the sensitivity of the IGRA may not be as high as expected in patients with active TB, and that patients with advanced HIV and severe immuno-suppression have an increased proportion of indeterminate results ([1-7] Aabye et al in prep). Alternative biomarkers expressed in higher amounts could prove less susceptible to immuno-suppression and could therefore be used in future developments of the IGRA tests. In this short communication we have compared the diagnostic potential of three novel biomarkers – CCL2/MCP-1, CCL7/MCP-3 and IL-1RA – with the current established biomarker IFN-γ and the newly described CXCL10/IP-10 and CCL8/MCP-2.

CXCL10 or Interferon induced protein (IP)-10 is a CXC chemokine mainly produced by monocytes and T cells. IP-10 is elevated in the serum of TB patients[8], and we have recently shown that IP-10 is produced in a highly antigen-dependent manner following *M. tuberculosis *antigen challenge[9]. We have shown that a diagnostic test for infection with *M. tuberculosis *using IP-10 performed comparably with a commercial IGRA in both active and latent TB disease ([10,11], Aabye et al in prep). CCL8 or monocyte chemoattractant protein (MCP)-2 is a CC chemokine that mainly attracts monocytes and T cells [12] and MCP-2 mRNA is upregulated in monocytes following infection by *M. tuberculosis*[13]. Similar to IP-10 we have recently demonstrated that MCP-2 expression is induced upon antigen challenge in the IGRA test assay, and that it holds diagnostic potential[11]. Interleukin 1 receptor antagonist (IL-1RA) is a natural antagonist of IL-1 produced by macrophages. IL-1RA has been suggested as a marker for TB disease activity and response to treatment[14]. CCL2 or MCP-1 is a CC chemokine which primarily targets monocytes and T cells[12,15]. MCP-1 is produced by PPD challenge in vitro [16] and serum levels have been associated with TB disease activity and treatment response([17], Heeland M, et al in prep). CCL7 or MCP-3 is produced by macrophages and attracts monocytes [12]. MCP-3 has been found elevated in bronchoalveolar lavage fluid and biopsy specimens of subjects with pulmonary tuberculosis[18].

## Methods

### Patient material

This study included eight patients with active tuberculosis and seven healthy unexposed controls. Four of the patients were from the study area of the Bandim Health Project in Guinea Bissau, West Africa of which all were diagnosed TB positive using sputum smear microscopy and/or clinical evaluation according to WHO guidelines. The remaining four TB patients were included from the department of infectious diseases at the Copenhagen University, Hvidovre Hospital all of which were sputum-smear-positive and culture positive. The seven healthy controls were recruited among junior doctors and students at Copenhagen University, Hvidovre Hospital. The median age of the TB patients was 44 (range 20–71) and 32 (28–34) for the controls. All TB patients were tested HIV negative; and all were included within the first two weeks of chemotherapy, median 0 days (range 0–14 days).

The study was approved by the Ethical Committee of Copenhagen and Frederiksberg Commune (KF01278477) and the Ethical Committee of Guinea Bissau.

### Whole blood stimulation

Briefly, 1 ml of blood was drawn directly into vaccutainer tubes from Cellestis (Carnegie, Australia). The tubes were precoated with saline (un-stimulated negative control), peptides of ESAT-6, CFP10 and TB 7.7 (the *M. tuberculosis *specific antigens), or PHA (positive mitogen control). The tubes were incubated for 20–24 hours at 37°C, and plasma was harvested and frozen until further analysis.

### Biomarker determination

The amount of IFN-γ produced was determined by ELISA using the manufacturer's instructions  and IFN-γ measurements were shown in pg/ml to facilitate the comparison with other biomarkers as described previously[9]. One International Unit (IU) of IFN-γ corresponds to 50 pg (NIBSC, Hertfordshire, UK). IP-10, MCP-1, MCP-2, MCP-3, and IL-1RA concentrations were measured by xMAP technology on the Luminex platform (Luminex Corporation), using Biosource reagents (Biosource, Camarillo, USA) acquired and analyzed with the StarStation v2.0 software (Applied Cytometry Systems) as described previously[9]. All measurements were performed in duplicates and blinded. In preliminary studies we had observed that the antigen and mitogen induced biomarker concentrations were above the upper limits of quantification (data not shown), thus measurements were performed at 1:8 dilution in assay diluent (Biosource) and concentrations were later multiplied with the dilution factor as recommended by the manufacturer (Biosource).

### Statistics

Concentration of IP-10, MCP-2, MCP-3, MCP-2, IL-1RA and IFN-γ were compared using Kruskal-Wallis test and Wilcoxon signed rank test. Data was analysed using SAS 9.1.3 (SAS institute, Cary, USA).

## Results

### Biomarker measurements

By screening 30 potential biomarkers (cytokines, chemokines, soluble receptors and receptor antagosnists) we found that IP-10, MCP-2, MCP-1, MCP-3 and IL-1RA were biomarkers expressed in high amounts upon challenge with *M. tuberculosis *antigens compared to un-stimulated levels (data not shown). Median and inter quartile range of biomarkers levels in un-stimulated, antigen-, and mitogen-stimulated whole blood from eight TB patients and seven controls are shown in table 1. All TB patients responded positive and all controls responded negative in the QFT-IT test.

**Table 1:** Plasma IP-10, MCP-3, MCP-2, IFN-γ, MCP-1 and IL-1RA release in antigen stimulated whole blood culture

	**Controls (n = 7)**	**TB patients (n = 8)**	**p-value**
**IP-10**			

Nil	5 (5–57)^1^	157 (102–301)	0.0047

Antigen	46 (0–63)	9010 (5542–13785)	0.0011

Mitogen	3601 (1702–5918)	4835 (2112–7075)	0.7285

**MCP-3**			

Nil	737 (320–1202)	3464 (1442–5517)	0.0372

Antigen	807 (147–1236)	9206 (7221–11296)	0.0012

Mitogen	2224 (1724–2964)	9393 (4538–13966)	0.0109

**MCP-2**			

Nil	24 (17–43)	26 (24–46)	0.4724

Antigen	33 (24–43)	2208 (914–3781)	0.0012

Mitogen	4673 (1908–6601)	3379 (474–5592)	0.4875

**IFN-γ**			

Nil	8 (7–9)	8 (7–14)	0.4859

Antigen	8 (6–9)	223 (179–601)	0.0012

Mitogen	1326 (534–1363)	327 (92–724)	0.0279

**MCP-1**			

Nil	6352 (3110–13560)	6120 (4723–15527)	0.8170

Antigen	7206 (5368–12471)	44991 (25831–56822)	0.0012

Mitogen	27773 (20500–42559)	46926 (26396–80328)	0.2030

**IL-1RA**			

Nil	3371 (1277–7983)	6328 (3035–9415)	0.4875

Antigen	3986 (2386–6893)	26002 (18226–33934)	0.0054

Mitogen	54397 (29233–77086)	15966 (12106–27296)	0.0091

^1 ^Median (Inter Quartile Range)

In whole blood cultures from all TB patients the antigens induced significant elevations in the levels of all 6 biomarkers compared to the controls (p < 0.006). The un-stimulated IP-10, MCP-2 and IFN-γ levels were significantly lower compared to MCP-1 (p < 0.0001), MCP-3 (p < 0.0001), and IL-1RA (p < 0.0001). But, un-stimulated IP-10 and MCP-3 levels among TB patients were significantly higher compared to controls (p < 0.04). Mitogen stimulation resulted in an increase in all biomarkers in both control and patient samples (p < 0.0001).

### Antigen-dependent biomarker production

**Figure 1:**
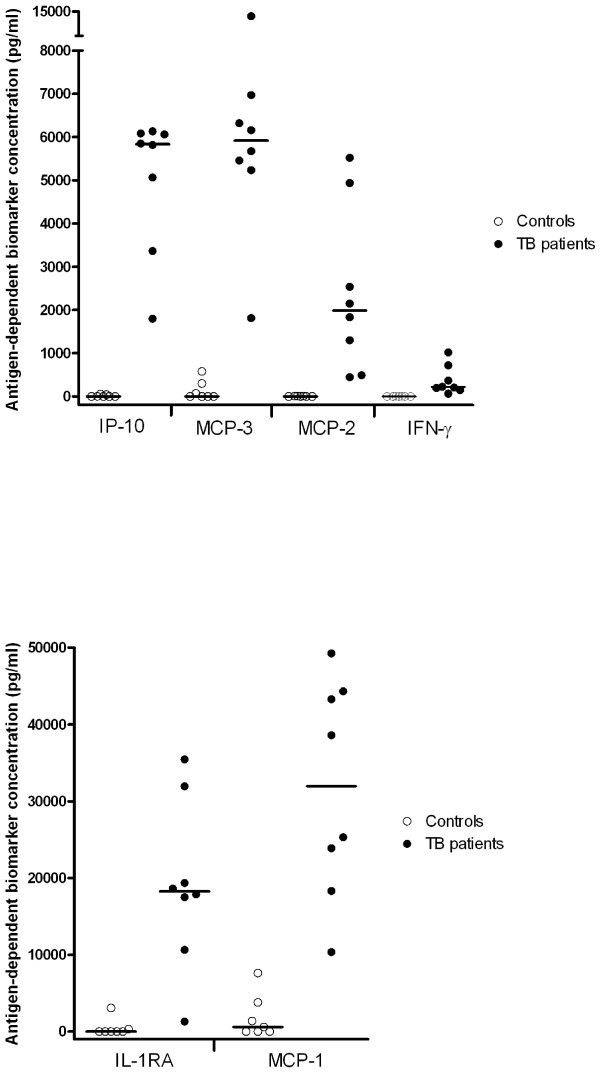
**Whole blood of eight patients with active TB, and seven healthy unexposed controls was stimulated 20 hours with either saline or *M. tuberculosis *specific antigens**. Biomarker production was measured in plasma supernatant by ELISA (IFN-γ) and multiplex technology (IP-10, MCP-3, MCP-2, IL-1RA and MCP-1). Antigen-dependent production represent the antigen stimulated sample subtracted the un-stimulated sample. Straight lines represent median values. Differences between healthy controls and patients were all significant p < 0.0001 (Kruskal-Wallis test).

The antigen-dependent biomarker expression was evaluated by subtracting the concentration in the un-stimulated tube from the levels measured in the antigen-stimulated tube. The individual measurements are depicted in figure 1a–b. The antigen-dependent biomarker levels were significantly higher in TB patients compared to controls (p < 0.006). For the TB patients the median antigen-dependent levels were: 31,962 pg/ml (range 10,354–49,276 pg/ml) for MCP-1, 18,254 pg/ml (1,297–35,458 pg/ml) for IL1-RA, 8,778 pg/ml (1,800–24,355 pg/ml) for IP-10, 5,918 pg/ml (1,812–13,658 pg/ml) for MCP-3 and 1,990 pg/ml (444–5,523 pg/ml) for MCP-2, and 215 pg/ml (64–1,018 pg/ml for INF-γ. The antigen-dependent IP-10, MCP-2, and IFN-γ concentrations in the controls were consistently low whereas one control responded with some antigen-dependent MCP-1, MCP-3 and IL-1RA production, another with some MCP-3 and IL-1RA production, and a third responded with some MCP-3 production (figure 1). IP-10, MCP-2, MCP-3, MCP-2 and IFN-γ, but not IL-1RA differentiated completely between patients and controls.

## Discussion

The ideal diagnostic biomarker gives a binary signal that clearly differentiates between healthy and infected individuals, and between different stages of disease e.g. latent and active disease. In the less perfect world of cellular assays for TB diagnosis, a good biomarker has low levels in un-stimulated samples, no response to antigen in samples from uninfected individuals, and strong responses to antigen in samples from patients. Till this date no biomarker can differentiate between active and latent TB. The two commercially available IGRA tests use IFN-γ as biomarker for TB infection. IFN-γ fulfils the criteria of low levels in un-stimulated samples and no responses to antigen in uninfected, but the levels expressed in response to antigens are low. To achieve maximum sensitivity, the cut off point for a positive IFN-γ test is set at the lowest possible level[19]. A recent meta-analysis estimated overall test sensitivity to be 70% – 90% and specificity 93 – 99% (depending on assay), and test performance is compromised in immuno-compromised individuals ([1,5], Aabye et al in prep)

This small proof of principle study compares three novel biomarkers for in-vitro diagnosis of TB with the current established biomarker IFN-γ and the newly identified IP-10 and MCP-2. The main new findings are that MCP-1, MCP-3, and IL-1RA all were induced in-vitro in response to antigen stimulation in significantly higher amounts in patients than in controls. All biomarkers were specifically induced after antigen-stimulation of whole blood from patients but not from controls, indicating a diagnostic potential. IP-10, MCP-3, and especially MCP-1, and IL-1RA were expressed in very high antigen-dependent levels in patients, but one patient had a low IL-1RA response. Some of the unexposed controls responded with antigen-dependent MCP-1, MCP-3, and IL-1RA production to the antigens, indicating that these biomarkers could be less specific. As seen in other studies the IP-10 levels were induced in higher levels upon antigen compared to mitogen stimulation[10,11]. This probably reflect that the PHA mitogen targets T cells and induce an IFN signal that downstream induces an IP-10 signal from the monocytes present in the sample. In contrast the antigen-dependent IP-10 signal is a result of a convergence of signals both from cell surface receptor interaction between the T cell and the Monocyte, and cytokine stimulation. Un-stimulated levels of IP-10 and MCP-3 were significantly higher in TB patients both from Denmark and from Guinea-Bissau compared with the levels seen in controls (table 1). We and others have observed this phenomenon for IP-10[8,9], but it has not been observed before for MCP-3. The levels of IP-10 present in the blood plasma at time of phlebotomy correlate to the levels of IP-10 seen in the un-stimulated sample (unpublished observation) and high levels appear to reflect severe infection[8]. Whether differences in un-stimulated levels of IP-10 and MCP-3 can be used in conjunction with antigen specific responses to differentiate active from latent TB disease, remains to be explored.

## Conclusion

In conclusion we have identified three novel potential *in-vitro *biomarkers for tuberculosis infection MCP-1, MCP-3 and IL-1RA. Of the biomarkers studied, MCP-2 and IP-10 held the most promise as the un-stimulated levels were low, and the antigen-simulated levels were high in patients and not in controls. Both biomarkers were expressed in much higher concentration compared to IFN-γ. Although the results of IP-10 and MCP-2 reproduce findings from more well powered studies the sample size is small and there is a risk of type 1 error. Further studies are needed to explore the potential of these novel biomarkers individually and in combination.

## Competing interests

Copenhagen University Hvidovre Hospital has applied for a patent disclosing IP-10 as marker for *M. tuberculosis *infection and one disclosing MCP-2 as marker for M tuberculosis infection. Morten Ruhwald, Pernille Ravn and Jesper Eugen-Olsen are registered as inventors on both pending applications. In 2006 Pernille Ravn was a consultant for Cellestis Ltd. to develop their clinical guidelines and received a single payment of €2000. The other authors have no conflicts of interest.

## Authors' contributions

MR, designed the study, included patients and controls in Guinea Bissau and Copenhagen, incubated the samples, did the immunoassays, analyzed the data and wrote the paper. MBA Supervised inclusion of patients and organized sample handling in Guinea Bissau. PAR, organized and supervised inclusion of patients in Guinea Bissau. JEO Supervised the lab work and co-designed the study. PER Co-designed the study, included patients in Copenhagen. All authors approved the final version of the paper.
